# Absence of Pannexin 1 Stabilizes Hippocampal Excitability After Intracerebral Treatment With Aβ (1-42) and Prevents LTP Deficits in Middle-Aged Mice

**DOI:** 10.3389/fnagi.2021.591735

**Published:** 2021-03-16

**Authors:** Nicolina Südkamp, Olena Shchyglo, Denise Manahan-Vaughan

**Affiliations:** ^1^Department of Neurophysiology, Medical Faculty, Ruhr University Bochum, Bochum, Germany; ^2^International Graduate School of Neuroscience, Ruhr University Bochum, Bochum, Germany

**Keywords:** pannexin 1 (Panx1), rodent, Alzheimer, beta-amyloid, CA1, hippocampus, LTP, amyloidosis

## Abstract

Beta-amyloid protein [Aβ(1-42)] plays an important role in the disease progress and pathophysiology of Alzheimer's disease (AD). Membrane properties and neuronal excitability are altered in the hippocampus of transgenic AD mouse models that overexpress amyloid precursor protein. Although gap junction hemichannels have been implicated in the early pathogenesis of AD, to what extent Pannexin channels contribute to Aβ(1-42)-mediated brain changes is not yet known. In this study we, therefore, investigated the involvement of Pannexin1 (Panx1) channels in Aβ-mediated changes of neuronal membrane properties and long-term potentiation (LTP) in an animal model of AD. We conducted whole-cell patch-clamp recordings in CA1 pyramidal neurons 1 week after intracerebroventricular treatments of adult wildtype (wt) and Panx1 knockout (Panx1-ko) mice with either oligomeric Aβ(1-42), or control peptide. Panx1-ko hippocampi treated with control peptide exhibited increased neuronal excitability compared to wt. In addition, action potential (AP) firing frequency was higher in control Panx1-ko slices compared to wt. Aβ-treatment reduced AP firing frequency in both cohorts. But in Aβ-treated wt mice, spike frequency adaptation was significantly enhanced, when compared to control wt and to Aβ-treated Panx1-ko mice. Assessment of hippocampal LTP revealed deficits in Aβ-treated wt compared to control wt. By contrast, Panx1-ko exhibited LTP that was equivalent to LTP in control ko hippocampi. Taken together, our data show that in the absence of Pannexin1, hippocampi are more resistant to the debilitating effects of oligomeric Aβ. Both Aβ-mediated impairments in spike frequency adaptation and in LTP that occur in wt animals, are ameliorated in Panx1-ko mice. These results suggest that Panx1 contributes to early changes in hippocampal neuronal and synaptic function that are triggered by oligomeric Aβ.

## Introduction

One key feature of the pathophysiology of Alzheimer's disease (AD) is beta-amyloid [Aβ(1-42)], a peptide that is a fundamental component of amyloid plaques: insoluble deposits of material within the brain that appear in the late stages of AD. Long before plaques may form, Aβ(1-42) is believed to play a key role in the onset and development of pre-symptomatic AD (Mucke et al., 2000; Fukumoto et al., 2010; Edwards, 2019). In particular, the oligomeric form of Aβ(1-42) causes debilitation of hippocampal long-term potentiation (LTP), even when topically applied to a hippocampal slice (Wang et al., 2004), or acutely applied *in vivo* (Klyubin et al., 2005). Acutely, oligomeric Aβ(1-42) elevates intracellular Ca^2+^-levels that impairs neuronal viability (Hardy and Higgins, 1992; Mattson et al., 1992; Kuchibhotla et al., 2008). It also triggers production of reactive oxygen species (Behl et al., 1994; Hensley et al., 1994) and activation of microglia (Combs et al., 1999), that also contribute to the pathophysiology of AD. Although the precise mechanisms whereby oligomeric Aβ mediates its effects are as yet unclear, changes occur rapidly once it is present in the brain. Intracerebral inoculation of healthy rats with oligomeric Aβ (1-42) triggers debilitations of hippocampal LTP that are accompanied by changes in neuronal oscillations (Kalweit et al., 2015). The latter effect may be supported by Pannexin1 (Panx1) channels shared by hippocampal neurons (Lopatár et al., 2015). Furthermore, degranulation of mast cells by Aβ(1-25) is mediated by Panx1 (Harcha et al., 2015), suggesting that the hemichannel plays an active role in the pathophysiology of AD.

Pannexins, including Panx1, Pannexin 2 and 3 are transmembrane large-conductance cation channels that enable the movement of ions and small molecules between intracellular and extracellular compartments of vertebrate cell systems (Sosinsky et al., 2011; Scemes and Velíšková, 2017; Beyer and Berthoud, 2018). Panx1-channels are ubiquitously expressed in all organs (Sandilos and Bayliss, 2012; Li et al., 2015) and have been described in the brain, in structures such as the cerebellum, thalamus and hippocampus (Scemes and Velíšková, 2017). Within the hippocampus they are expressed on both neurones and interneurones (Zoidl et al., 2007), as well as on astrocytes and microglia (Giaume et al., 2017; Willebrords et al., 2017). They have been subjected to particular scrutiny in the hippocampal CA1 region, where high levels have been reported on pyramidal cells (Ray et al., 2005). Localized expression on the postsynaptic membrane has also been described (Zoidl et al., 2007; Sosinsky et al., 2011).

Panx1-channels are activated by neuronal depolarization, but can also be opened by mechanical stress (Bao et al., 2004), elevated intracellular Ca^2+^, K^+^ or ATP levels, or by hypoxia (Locovei et al., 2006). Stimulation of purinergic P2X- oder P2Y-receptors (Locovei et al., 2006) or glutamatergic N-methyl-D-aspartate receptors (NMDAR) (Scemes et al., 2009; Dahl and Keane, 2012; Sandilos and Bayliss, 2012; Penuela et al., 2013; Chiu et al., 2014; Scemes and Velíšková, 2017; Willebrords et al., 2017; Dahl, 2018) also results in activation of Panx1-channels. The channels can undergo conformational changes that result in alterations of their permeability, such as enabling selective passage of Cl^−^ (Dahl, 2018; Wang and Dahl, 2018). The channels are Ca^2+^-permeable and support the propagation of Ca^2+^-waves (Scemes et al., 2009; Dahl and Keane, 2012; Penuela et al., 2013; Scemes and Velíšková, 2017).

Under physiological conditions, Panx1-channels support communication between neurons and glia and also play a role in immune responses (Scemes and Velíšková, 2017). In addition to their contribution to neuronal excitability (Scemes and Velíšková, 2017), the channels contribute to synaptic plasticity (Prochnow et al., 2012; Ardiles et al., 2014), hippocampus-dependent learning and memory (Prochnow et al., 2012), and the regulation of the sleep-waking cycle (Kovalzon et al., 2017). Panx1 acts as a tumor suppressor in glioma (Sandilos and Bayliss, 2012) and various roles in neuroprotection and homeostasis have been described (Prochnow et al., 2012; Freitas-Andrade and Naus, 2016; Dahl, 2018). Given this plethora of roles, it is therefore perhaps not too surprising that dysfunctions of Panx1-channels lead to pathophysiological changes of brain function. Panx1 activation by ATP released from dying cells serves to enhance ATP release and thus, further boost apoptosis (Chekeni et al., 2010; Cisneros-Mejorado et al., 2015; Crespo Yanguas et al., 2017). A similar process takes place with regard to neuroinflammation, whereby elevated intracellular levels of Ca^2+^, K^+^ or ATP stimulate the channels, which then, supported by purinergic P2X7-receptor activation, lead to activation of inflammasomes, the release of cytokines and to cell death (Silverman et al., 2009; Adamson and Leitinger, 2014; Makarenkova and Shestopalov, 2014; Crespo Yanguas et al., 2017). These latter processes are believed to form the basis of Panx1-channel contributions to the pathophysiology of AD (Giaume et al., 2017). Notably, Aβ leads to a prolonged opening of Panx1-channels (Orellana et al., 2011; Quintanilla et al., 2012; Freitas-Andrade and Naus, 2016). Furthermore, the hemichannel's ability to promote inflammation supports disease progress (Harcha et al., 2015) and P2X7-mediated retinal degeneration triggered by Aβ (Olivier et al., 2016). These observations have to the proposal that Panx1 could be targeted to prevent inflammasome activation in brain disease (Jian et al., 2016; Kim et al., 2017). As yet, the contribution of Panx1 to Aβ(1-42)-mediated debilitations of the hippocampus, a key target of AD, has not been investigated, however.

In this study, we explored the cellular mechanisms underlying the putative contribution of Panx1 to AD. Thus, we explored to what extent Panx1 knockout alters the vulnerability of the hippocampus to the oligomeric form of Aβ(1-42). Here, Aβ(1-42), was applied intracerebrally to Panx1-ko, or wildtype, mice 1 week before *in vitro* examination of neuronal properties and synaptic plasticity. Consistent with reports by others, we observed elevated neuronal excitability and enhanced LTP in the hippocampus of Panx1-ko mice compared to wildtype controls (Prochnow et al., 2012) after treatment with control peptide. Patch clamp recordings also revealed that firing frequency was significantly decreased in both groups. Furthermore, wild-type hippocampi exhibited Aβ-mediated impairments in LTP and spike frequency adaptation that were absent in Panx1-ko hippocampi. These findings suggest that in the early stages of Aβ-mediated hippocampal debilitation, suppression of Panx1 hemichannels could prove beneficial to hippocampal function.

## Materials and Methods

### Animals

All experiments were conducted using 4–10 month-old male and female Panx-1LoxP/CMV-cre mice and their wildtype littermates (Zentrale Versuchstierhaltung Medizin, Ruhr University Bochum). Animals were housed in custom-made acclimatized and ventilated holding cupboards in an animal-housing room with a controlled 12-h light/dark cycle. Water and food were available *ad libitum*. The study was carried out in accordance with the European Communities Council Directive of September 22nd, 2010 (2010/63/EU) for care of laboratory animals and all experiments were conducted according to the guidelines of the German Animal Protection Law. They were approved in advance by the North Rhine-Westphalia (NRW) State Authority (Landesamt für Arbeitsschutz, Naturschutz, Umweltschutz und Verbraucherschutz, NRW). All efforts were made to minimize the number of animals used.

### Treatment With Aβ(1-42)

Oligomeric Aβ(1-42) was prepared and aggregated as described before (Kalweit et al., 2015). The soluble Aβ(1-42) peptide was prepared in phosphate-buffered saline at p.H. 7.4. It was subsequently diluted to a concentration of 50 μM, shock-frozen with liquid nitrogen and stored at −80°C. The Aβ solution was incubated for 3 h 1 day before the experiment to ensure that oligomerization occurred (Kalweit et al., 2015) and subsequently stored at −80°C. It was thawed at room temperature 5 min before application in a dose of 10 μM and a volume of 1 μl to both lateral cerebral ventricles of anesthetized mice by means of a Hamilton syringe. This volume differed from that one used in the Kalweit study that involved intracerebral treatment of *rats* (with 5 microliter in a dose of 10 μM) (Kalweit et al., 2015). Control animals received scrambled Aβ –peptide (Yamin et al., 2016) in a dose of 10 μM in a volume of 1 μl. The sequence of the scrambled peptide (Biotrend Ag Zurich, Catalog No.: BP0028-scr) was H-Ala-Ile-Ala-Glu-Gly-Asp-Ser-His-Val-Leu-Lys-Glu-Gly-Ala-Tyr-Met-Glu-Ile-Phe-Asp-Val-Gln-Gly-His-Val-Phe-Gly-Gly-Lys-Ile-Phe-Arg-Val-Val-Asp-Leu-Gly-Ser-His-Asn-Val-Ala-OH. Experiments were also conducted in the absence of any treatments to verify that the control peptide did not elicit independent effects on neuronal responses (see results section).

### Slice Preparation

Animals were deeply anesthetized with isoflurane before decapitation. Immediately afterwards, 350 μm sagittal hippocampal slices were prepared in cold (1–4°C), oxygenated saccharose solution (in mM: 87 NaCl, 2.6 MgSO_4_, 75 Saccharose, 2.5 KCl, 1.25 NaH_2_PO_4_, 26 NaHCO_3_, 0.5 CaCl_2_, 2 D-Glucose) (95% O_2_, 5% CO_2_). Slices were then incubated in the holding chamber with aCSF (in mM: 125 NaCl, 3 KCl, 2.5 CaCl_2_, 1.3 MgSO_4_, 1.25 NaH_2_PO_4_, 26 NaHCO_3_ and 13 D-Glucose) at a constant flow rate of 2 ml/min at 30°C for at least 30 min before recordings were commenced.

### Patch Clamp Recordings

**Figure 1:**
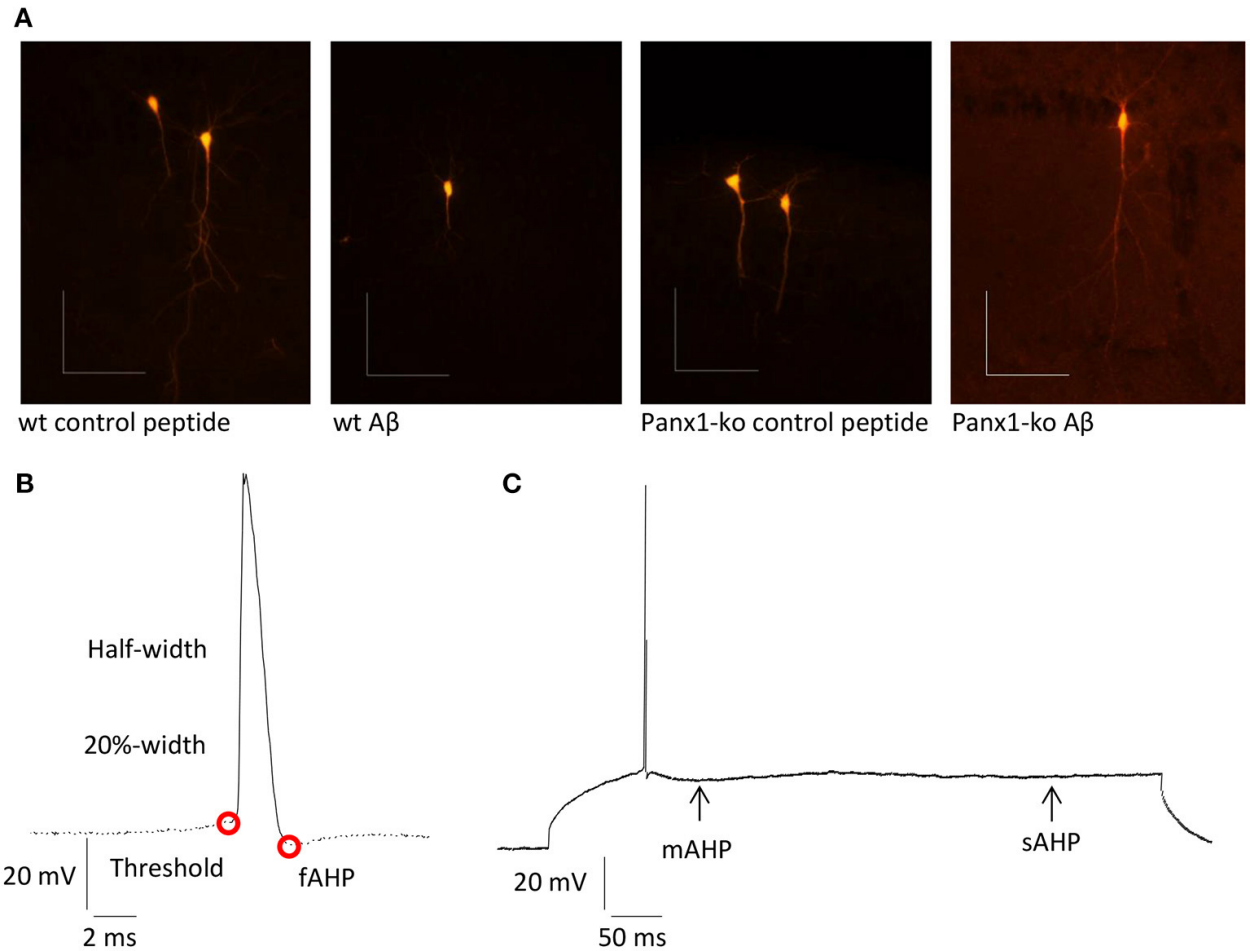
**(A)** Examples of pyramidal cells from wt and ko hippocampi that were filled with biocytin after recordings. Scale bars: horizontal 0.1 mm, vertical 0.1 mm. **(B)** Summary of neuronal properties assessed within the action potential (AP). Red circles show the AP threshold and fast afterhyperpolarization (fAHP). **(C)** Medium (mAHP) and slow elements of the AHP (sAHP) are indicated by the arrows.

Patch clamp recordings were conducted as described previously (Novkovic et al., 2015). Hippocampal slices were placed in a recording chamber that was located on an upright microscope. Slices were continuously perfused with oxygenated aCSF (at a constant flow rate of 1 – 2 ml/min). Recording pipettes were pulled from borosilicate glass pipettes (1.5 mm external diameter) with a resistance of 6-10 MΩ and were then filled with intracellular solution (in mM: 97.5 potassium gluconate, 32.5 KCl, 5 EGTA, 10 Hepes, 1 MgCl_2_, 4 Na_2_ ATP, adjusted to pH 7.3 with KOH). Recordings were performed from visually identified soma of pyramidal neurons in the CA1 region. To verify the identity of the pyramidal cells, biocytin-filling of cells was conducted after recordings in the following manner (Figure 1A): Biocytin (1 mg/ml) was added to the intracellular solution. No gross morphological changes to the cells were evident in Panx1-ko, or after Aβ-treatment. After patch clamp recordings, slices were fixed in 4% paraformaldehyde in phosphate buffered saline (PBS) at 4°C for at least 48 h. Afterwards, the slices were kept in 30% saccharose solution for at least 7 days. After slicing into slices of 30 μm at −35°C, they were incubated overnight at room temperature with streptavidin 1:1000 (Cy™3-conjugated Streptavidin, Dianova, Hamburg, Germany) in 25% 0.2% Tx-PBS (Triton X-100), 75% PBS and bovine serum albumin. Slices were preserved in the dark and examined with a fluorescence microscope (Leica DFC300 FX, Leica Microsystems GmbH, Wetzlar, Germany).

Intrinsic membrane properties were determined by means of a HEKA EPC10 amplifier using the PATCHMASTER acquisition software (HEKA Elektronik Dr. Schulze GmbH, Lambrecht/Pfalz, Germany). The following properties were assessed: resting membrane potential, input resistance, membrane time constant, excitatory threshold, sag, sag ratio, firing frequency, action potential (AP) threshold, spike amplitude, AP peak, half-width, 20%-width, time-to-peak, afterhyperpolarization (AHP), time peak to AHP (Figure 1B). In addition, fast, medium and slow elements of the AHP were determined (Figure 1C).

Data underwent low-pass filtering at 2.9 kHz and were digitized at 10 kHz. FITMASTER software (HEKA Elektronik Dr. Schulze GmbH, Lambrecht/Pfalz, Germany) was used for offline data analysis. Input resistance was calculated from the slope of the linear fit of the relationship between the change in membrane potential (ΔV) and the intensity of the injected current (between−120 pA and +90 pA). The time constant was determined from an exponential fit of the averaged voltage decay. The resting membrane potential was determined from the mean of 30 s basal recording time. The minimum current needed to induce an action potential was defined as the threshold current. The action potential amplitude was measured as the voltage difference between the threshold and the peak. Firing properties were investigated by applying current steps of Δ50 pA in hyperpolarizing and depolarizing square pulses (1 s duration) through the patch-clamp electrode (in the range of−300–400 pA).

Spike frequency adaptation was determined by counting the number of spikes separately during each 100 ms of the 1 s depolarizing square pulse of 300 pA and converting the number into a frequency in Hz.

### fEPSP Recordings and Induction of LTP

A bipolar stimulation electrode (Fredrick Haer, Bowdowinham, ME, USA) was placed in the Stratum radiatum of the CA1 region of the hippocampus and a glass field recording electrode (impedance: 1–2 MΩ) filled with aCSF was positioned in the CA1 dendritic area.

Test-pulse stimuli (0.025 Hz, 0.2 ms duration) were applied to evoke field excitatory post-synaptic potentials (fEPSPs) that were recorded with a sample rate of 10,000 Hz. For each time point, five responses were averaged. Before recordings were started, a stimulus-response relationship was obtained with a stimulation range of 50–600 μA (in 50 μA steps) to detect the maximal fEPSP. The stimulation strength used for test-pulses was the stimulus intensity that evoked ca. 50% of the maximal fEPSP. After recording basal synaptic transmission for 40 min, LTP was induced by using theta burst stimulation (TBS) with three trains 10 s apart, each consists of 10 bursts of four pulses each at 100 Hz, delivered 200 ms apart (Novkovic et al., 2015).

### Statistical Analysis

A one way analysis of variance (ANOVA) was used for statistical analysis, with the exception of analysis of firing frequency and spike frequency adaptation, where an ANOVA with repeated measures was used. Where appropriate, a *post-hoc* Student's t-test or Fischer's least significant difference (LSD) test was used to determine if statistical significances occurred between two individual test conditions. Data are expressed as the mean ± standard error of the mean. “N” signifies the number of animals and “n” signifies the number of hippocampal slices (LTP experiments), or cells (for patch clamp data).

## Results

### Panx1 Knockout Mice Exhibit Higher Excitability Compared to Wild-Type Mice

**Figure 2:**
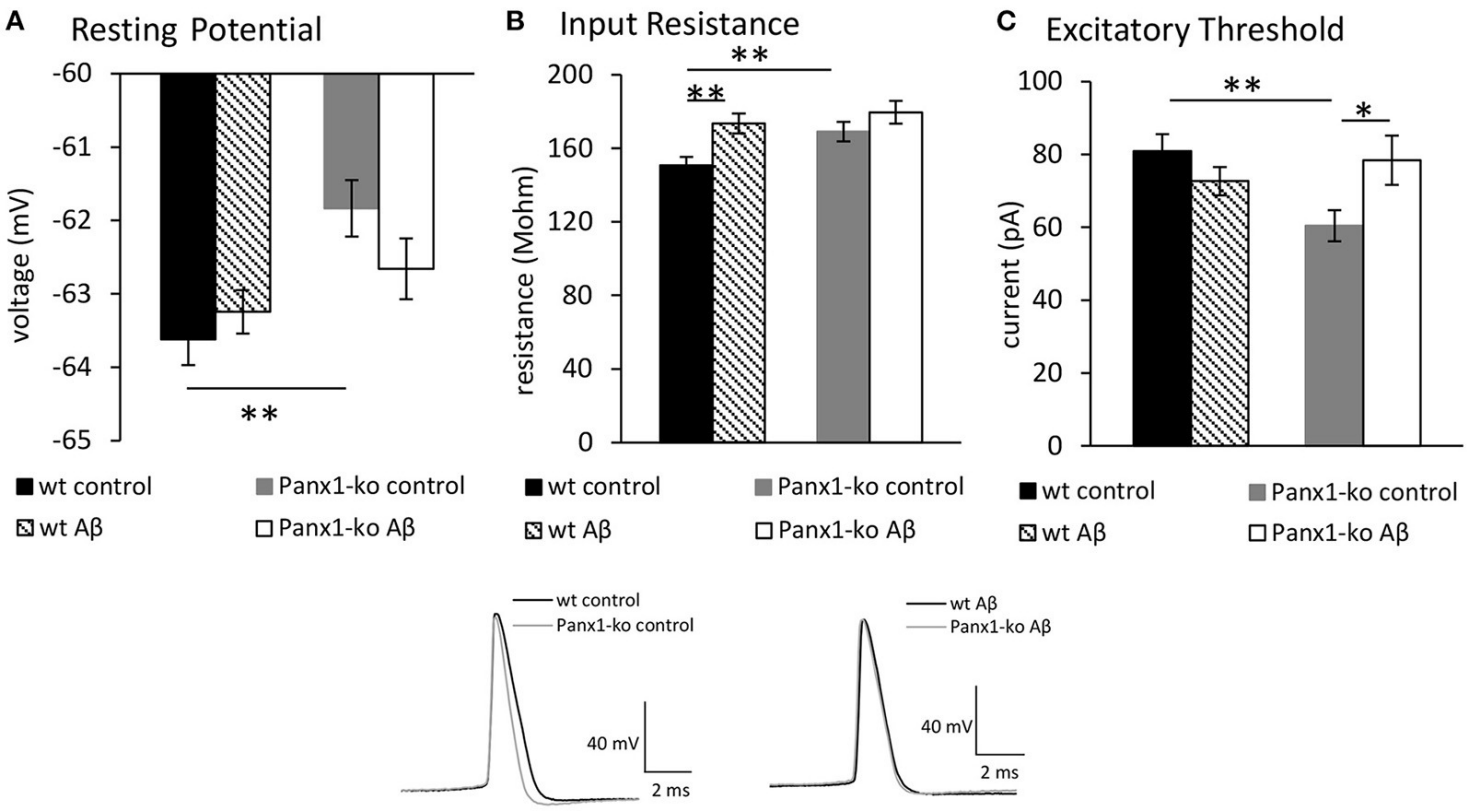
Panx1 knockout mice exhibit higher excitability compared to wild type mice. Aβ-treatment has selective effects on excitability in both Panx1-ko and wild-type hippocampi. In control (scrambled peptide-treated) animals, the hippocampi of Panx1-ko mice showed a more positive resting (membrane) potential **(A)**, a higher input resistance **(B)**, and a lower excitatory threshold **(C)** compared to control wild-type (wt) hippocampi. One week following intracerebral treatment of mice with oligomeric Aβ(1-42) peptide, wt and Panx1-ko hippocampi exhibited unchanged resting (membrane) potentials **(A)**. Input resistance was signficantly increased in Aβ-treated wt and unchanged in Panx1-ko hippocampi **(B)**. The increase in input resistance in wt was equivalent to input resistance detected in Panx1-ko. The excitatory threshold was unchanged in Aβ -treated wt and increased in Panx1-ko **(C)**. *p < 0.05, **p < 0.01. Insets: Analog examples of action potentials evoked in control peptide-treated wt and Panx1-ko (left) and in Aβ -treated animals (right).

We first compared excitability properties in Panx1-ko (*N* = 6, *n* = 34) and wt mice (*N* = 6, *n* = 34) 1 week after treatment with control (scrambled Aβ) peptide. We observed that the resting membrane potential (Figure 2A) was less negative and the input resistance (Figure 2B) was significantly higher in Panx1-ko compared to wt hippocampi (Table 1A). Furthermore, the excitatory threshold was lower (Figure 2C, Table 1A).

**Table 1:** Passive and active neuronal properties in wild-type vs. Panx1-ko hippocampi after treatment with control peptide.

**(A) The table shows passive und active neuronal properties in wild-type (wt) and ko Panx1 mice following treatment with control (scrambled) peptide. Firing frequencies (FF) evoked with currents in the range of 50 though 400pA are shown**			
	**Wt**	**Panx1-ko**	***t*****-test**^**$**^**/ ANOVA**
Resting potential (mV)	−63.62 ± 0.35	−61.84 ± 0.38	***p*** **=** **0.0018**^**$**^
Input resistance (MΩ)	150.79 ± 4.55	169.1 ± 5.32	***p*** **=** **0.008**^**$**^
Membrane time constant (ms)	16.34 ± 0.73	15.48 ± 1.01	*p* = 0.5^**$**^
Excitatory threshold (pA)	80.88 ± 4.74	60.44 ± 4.29	***p*** **=** **0.002**^**$**^
FF 50 pA	0.12 ± 0.07	2.26 ± 0.76	ANOVA, firing frequency: ***F***_**(3, 132)**_ **=** **7.98**, ***p*** **=** **0.00006**
FF 100 pA	4.56 ± 0.72	8.82 ± 1.57	
FF 150 pA	11.32 ± 0.78	14.76 ± 1.55	
FF 200 pA	14.62 ± 0.75	18.47 ± 1.45	
FF 250 pA	16.76 ± 0.80	20.62 ± 1.45	
FF 300 pA	18 ± 0.85	22.29 ± 1.45	
FF 350 pA	18.88 ± 0.91	22.44 ± 1.53	
FF 400 pA	19.56 ± 0.97	22.53 ± 1.55	
Sag (mV)	−11.46 ± 0.52	−12.82 ± 0.79	*p* = 0.15^**$**^
Sag ratio	0.90 ± 0.004	0.89 ± 0.006	*p* = 0.24^**$**^
**(B) The table shows action potential properties of wt- und Panx1-ko mice after control peptide treatment. Action potential (AP), afterhyperpolarization (AHP)**			
	**Wt**	**Panx1-ko**	***t*****-test**
AP threshold (mV)	−38.26 ± 0.89	−37.01 ± 1.06	*p* = 0.39
Spike amplitude (mV)	96.52 ± 0.91	97.00 ± 1.36	*p* = 0.74
Time to peak (ms)	0.43 ± 0.01	0.39 ± 0.01	***p*** **=** **0.0008**
Time peak to AHP (ms)	2.60 ± 0.06	2.29 ± 0.06	***p*** **=** **0.002**
Total spike time (ms)	3.03 ± 0.07	2.68 ± 0.06	***p*** **=** **0.001**
Ascending slope (mV/ms)	231.46 ± 7.44	254.56 ± 8.20	***p*** **=** **0.0081**
Descending slope (mV/ms)	41.08 ± 1.18	48.60 ± 1.37	***p*** **=** **0.0003**
Half-width (ms)	0.97 ± 0.02	0.86 ± 0.02	***p*** **=** **0.0008**
20%-width (ms)	1.45 ± 0.03	1.28 ± 0.03	***p*** **=** **0.0009**
AP peak (mV)	58.26 ± 0.49	59.99 ± 0.68	***p*** **=** **0.036**

*Significant effects are highlighted in bold. Values annotated with a $ refer to t-test*.

### Control Peptide-Treated Panx1 Knockout Mice Exhibit Reduced Spike Frequency Adaptation and Higher Action Potential Firing Frequency Compared to Control Wild-Type Mice

**Figure 3:**
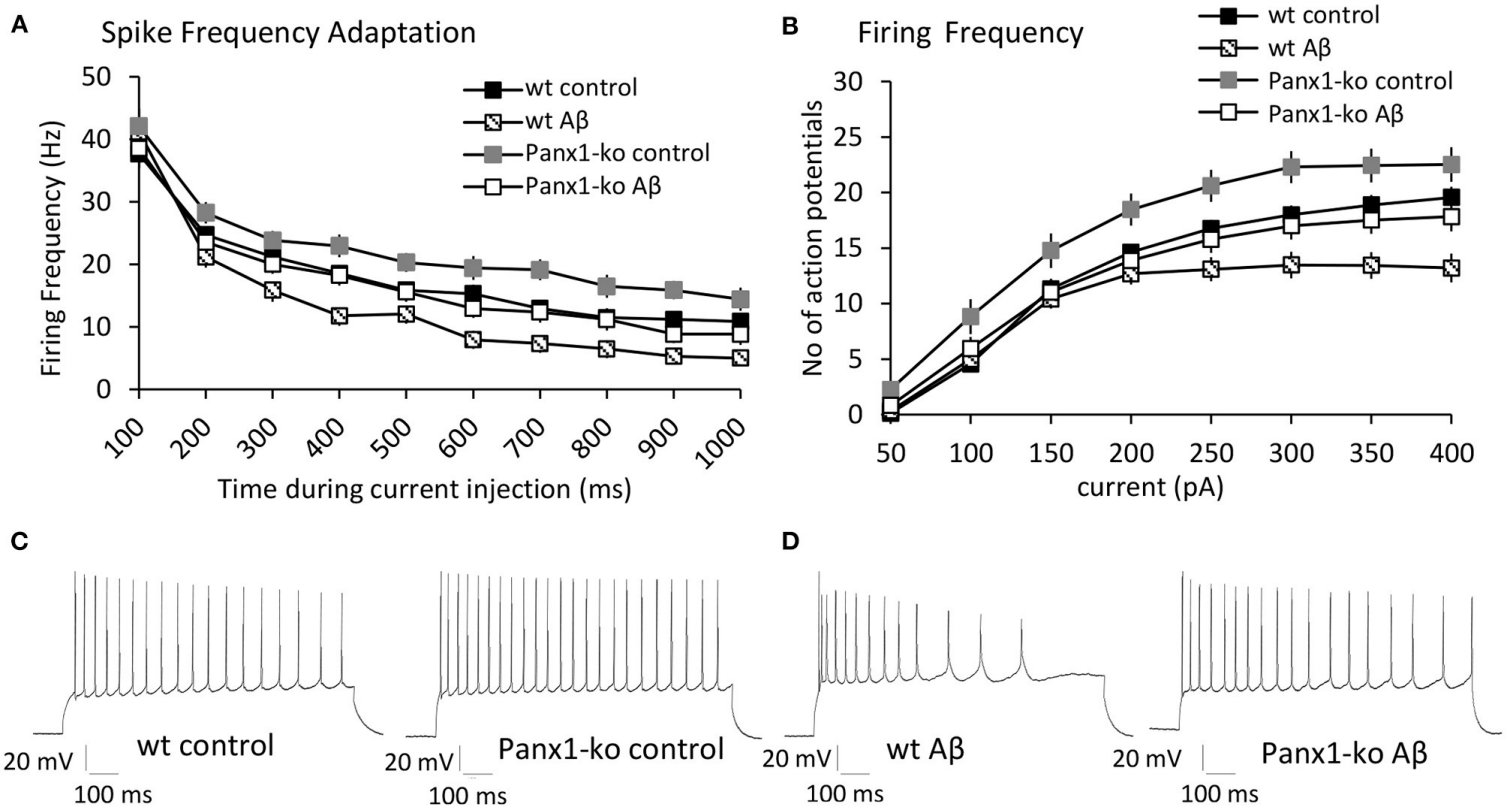
Panx1-knockout mice exhibit reduced spike frequency adaptation and higher action potential firing frequency compared to wild type mice after control-peptide treatment. Aβ-treatment accelerates spike frequency adaptation and reduces action potential firing frequency in both Panx1-ko and wild-type hippocampi. **(A)** Spike frequency adaptation (SFA) exhibited a more rapid adaptation in wt compared to Panx1-ko mice following current injections of 300 pA under control (scrambled peptide-treated) conditions. A comparison of SFA in Aβ-treated vs. control-treated wt revealed a significantly faster SFA in Aβ-treated wt hippocampi. SFA is equivalent in Aβ-treated Panx1-ko and wt control mice. **(B)** Action potential firing frequency was faster in Panx1-ko at all current steps, with the exception of 50 and 400 pA. Firing frequency was significantly decreased in both Aβ-treated wt. When all conditions were compared, control peptide-treated Panx1-ko showed the highest firing frequency that was significantly different from firing frequency in control peptide-treated wt. The decrease in firing frequency in Aβ-treated Panx1-ko hippocampi was indistinguishable from the firing frequency profile of control peptide-treated wt mice. Insets: analog examples of action potential trains by a current intensity of 300 pA in control peptide-treated wt and Panx1-ko mice **(C)** and in Aβ-treated wt and Panx1-ko mice **(D)**.

Reduced intrinsic excitability is associated with enhanced spike-frequency adaptation (Disterhoft and Oh, 2007). We therefore wondered whether the increase of intrinsic excitability that we observed in Panx1-ko mice is accompanied by reduced SFA and whether this is modulated by Aβ-treatment. Analysis of spike frequency adaptation (SFA) revealed a more rapid adaptation in wt compared to Panx1-ko mice following current injections of 300 pA under control (scrambled peptide-treated) conditions (Figures 3A,C, Table 1A). Thus, the enhanced intrinsic excitability in control Panx1-ko was indeed associated with poorer SFA.

Perhaps not surprisingly, the action potential firing frequency was faster in Panx1-ko at all current steps (*post-hoc* LSD *p* < 0.05 for all currents), with the exception of 50 and 400 pA (Figures 3B,C, Table 1A).

**Figure 4:**
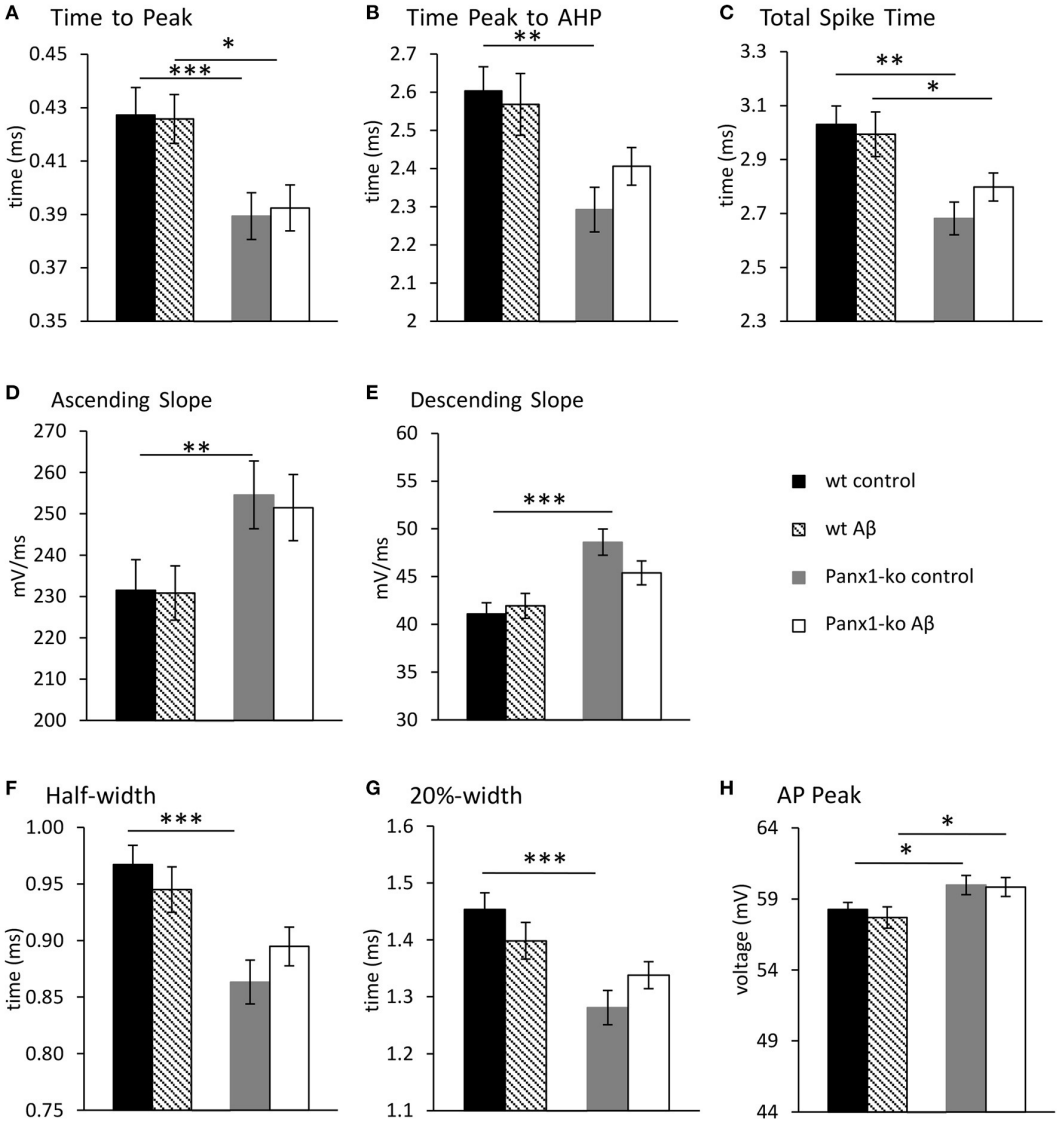
Action potential properties are unaltered in wild-type and Panx1-knockout hippocampus 1 week after Aβ-treatment. Under control (scrambled) peptide, conditions, the hippocampi of Panx-ko mice exhibited a faster time to peak of the action potential (AP) **(A)**, a faster time to peak of the afterhyperpolarization (AHP) **(B)** and a faster total spike time **(C)** compared to wt slices. The AP also exhibited a faster ascending slope **(D)** and a faster AP descending slope **(E)**, compared to wt. AP half-width **(F)** and 20%-width **(G)** were reached more quickly and the AP peak **(H)** was significantly larger in panx1-ko hippocampus compared to hippocampi from wt-animals. One week after intracerebral treatment with Aβ, the hippocampi of Panx-ko mice exhibited a faster time to peak of the action potential (AP) **(A)**, but differences in the time to peak of the afterhyperpolarization (AHP) **(B)** were not significant compared to wt slices. Panx-ko hippocampi exhibited a faster total spike time **(C)** compared to wt slices, but differences in the ascending AP slope **(D)** and the descending AP slope **(E)** were not significant compared to wt hippocampi. AP half-width **(G)** and 20%-width **(G)** were equivalent in Aβ-treated Panx1-ko and wt, but the AP peak was significantly larger in Aβ-treated Panx1-ko hippocampi compared to Aβ,-treated hippocampi from wt-animals **(H)**. No specific effects of Aβtreatment were found compared to control peptide-treatment when these conditions were compared in Panx1-ko mice, or compared in wt mice. *p < 0.05, **p < 0.01, ***p < 0.001.

When we examined additional action potential properties in Panx1-ko mice, we observed that the time-to-peak was shorter (Figure 4A, Table 1B), and the repolarisation time was faster (Figure 4B, Table 1B). The total spike time was decreased in Panx1-ko (Figure 4C, Table 1B), and the ascending and descending slope of the AP were increased (Figures 4D,E, Table 1B). In addition, the half-width and 20% width of the AP were decreased (Figures 4F,G) and the AP peak was increased (Figure 4H, Table 1B).

### The Fast Afterhyperpolarization Is Increased in Control Panx1 Knockout Mice

**Figure 5:**
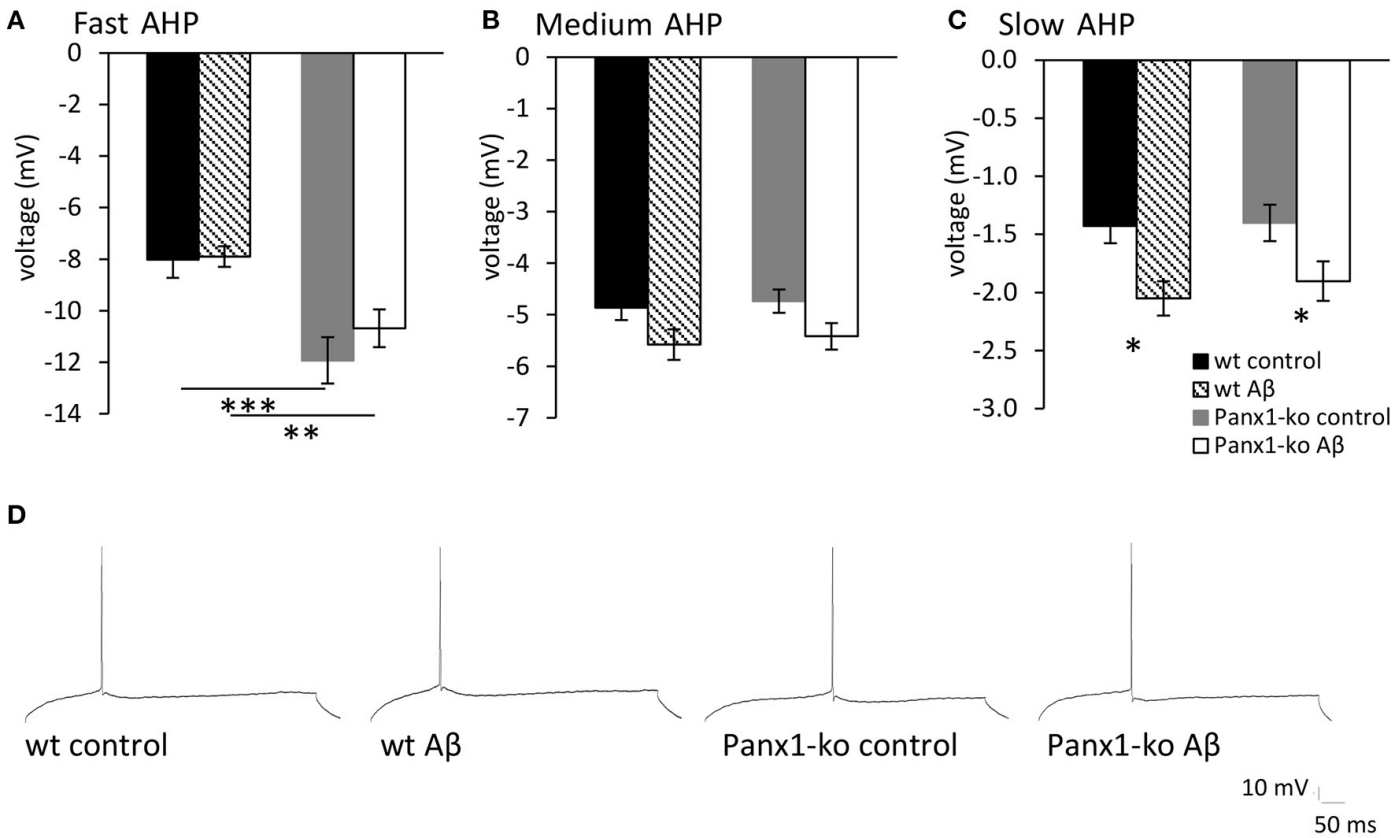
The afterhyperpolarization is altered in control peptide and Aβ-treated wild-type and Panx1-knockout hippocampus. **(A)** In control (scrambled peptide-treated) wt and Panx1-ko animals, the fast afterhypopolarization (AHP) was significantly more negative in Panx1-ko hippocampi compared to wt. One week following Aβ-treatment this difference was also present. **(B)** The medium AHP was equivalent in Panx1-ko and wt hippocampi under all conditions tested. **(C)** The slow AHP was equivalent in wt and Panx1-ko hippocampi following control peptide-treatment. Both wt and Panx1-ko hippocampi exhibited a significantly increased negativity in the slow AHP after Aβ-treatment. Effects were equivalent in wt and Panx1-ko. *p < 0.05, **p < 0.01, ***p < 0.001. **(D)** Analog examples of AHP responses evoked in control and Aβ-treated wt and Panx1-ko hippocampi.

In general, the size of the afterhyperpolarization (AHP) is determined by the number of action potentials during a burst of activity and the corresponding elevation in intracellular Ca^2+^−levels (Disterhoft and Oh, 2007). In line with this, we observed that the fast AHP was enhanced in control (scrambled peptide-treated) Panx1-ko compared to wild-type controls (Figure 5, ^***^p < 0.001). By contrast, the medium and slow AHPs were not significantly different from one another in control wt and Panx1-ko hippocampi (Figure 5).

Taken together, both passive and active membrane properties revealed higher excitability in the hippocampi of control peptide-treated Panx1-ko mice compared to control peptide-treated wt mice.

### Differences in Neuronal Responses in Panx1 Knockout and Wild-Type Mice Are Not Caused by the Control Peptide Treatment

To clarify if the differences in responses between wt and ko mice were intrinsic, or resulted from treatment with control (scrambled) peptide, we compared control peptide effects with responses recorded in age-matched untreated animals (Table 2). Equivalent differences were detected in untreated wt (*N* = 6, *n* = 23) and Panx1-ko animals (*N* = 6, *n* = 19), as had been detected in animals that had been treated with scrambled peptide (wt *N* = 6, *n* = 34, Panx1-ko *N* = 6, *n* = 34). Here, resting membrane potential was comparable in wt mice and Panx1-ko responses were also equivalent (Tables 2A,B). Spike threshold was also comparable (wt:−38.26 mV control peptide vs.−37.03 untreated, *p* = 0.39; Panx1-ko:−39.02 control peptide vs.−37.01 untreated, *p* = 0.23). Firing frequency was also comparable in control peptide-treated and untreated wt or Panx1-ko mice (Tables 2A,B), whereby the higher firing frequency of Panx1-ko compared to wt mice was still evident in scrambled peptide-treated hippocampi [ANOVA: *F*_(1, 40)_ = 4.9812, *p* = 0.03] (Supplementary Figure 1).

**Table 2:** Comparison of passive and active neuronal properties in untreated and scrambled peptide-treated wild-type and Panx1-ko hippocampi.

**(A)**			
	**wt untreated**	**wt scr Aβ**	***t*****-test/ANOVA**
Resting membrane potential (mV)	−62.95 ± 0.56	−63.62 ± 0.35	0.29
FF 50 pA	0 ± 0	0.12 ± 0.07	ANOVA Firing Frequency: *F*_(1, 54)_ = 0.004, *p* = 0.95
FF 100 pA	3.74 ± 0.86	4.56 ± 0.72	
FF 150 pA	9.91 ± 1.42	11.32 ± 0.78	
FF 200 pA	13.96 ± 1.55	14.62 ± 0.75	
FF 250 pA	16.65 ± 1.72	16.76 ± 0.80	
FF 300 pA	18.78 ± 1.85	18 ± 0.85	
FF 350 pA	19.96 ± 1.88	18.82 ± 0.93	
FF 400 pA	21.30 ± 1.93	19.56 ± 0.97	
Threshold (mV)	−37.03 ± 1.15	−38.26 ± 0.89	0.39
Peak (mV)	58.21 ± 0.74	58.26 ± 0.49	0.95
Spike Amp (mV)	95.24 ± 1.36	96.52 ± 0.91	0.42
Time to peak (ms)	0.40 ± 0.01	0.43 ± 0.01	0.12
AHP min (mV)	−46.91 ± 0.68	−46.27 ± 0.48	0.44
AHP depth (mV)	9.88 ± 0.76	8.01 ± 0.72	0.09
Peak to AHP (ms)	2.45 ± 0.08	2.6 ± 0.06	0.13
Total spike time (ms)	2.85 ± 0.08	3.03 ± 0.07	0.1
**(B)**			
	**panx1-ko untreated**	**panx1-ko scr Aβ**	***t*****-test/ANOVA**
Resting membrane potential (mV)	−61.38 ± 0.57	−61.84 ± 0.39	0.5
FF 50 pA	1.26 ± 0.80	2.26 ± 0.77	ANOVA Firing Frequency: *F*_(1, 51)_ = 0.19, *p* = 0.67
FF 100 pA	7.53 ± 1.59	8.82 ± 1.59	
FF 150 pA	14.32 ± 1.97	14.76 ± 1.57	
FF 200 pA	18.89 ± 2.01	18.47 ± 1.47	
FF 250 pA	22.05 ± 2.09	20.61 ± 1.47	
FF 300 pA	24.21 ± 2.02	22.29 ± 1.47	
FF 350 pA	25.47 ± 2.02	22.44 ± 1.56	
FF 400 pA	25.58 ± 2.23	22.53 ± 1.58	
Threshold (mV)	−39.02 ± 1.18	−37.01 ± 1.06	0.23
Peak (mV)	57.84 ± 1.38	59.99 ± 0.68	0.13
Spike Amp (mV)	96.86 ± 1.80	97.00 ± 1.36	0.95
Time to peak (ms)	0.41 ± 0.01	0.39 ± 0.01	0.09
AHP min (mV)	−48.82 ± 0.85	−48.94 ± 0.58	0.9
AHP depth (mV)	9.80 ± 0.64	11.93 ± 0.90	0.095
Peak to AHP (ms)	2.55 ± 0.35	2.29 ± 0.06	0.37
Total spike time (ms)	2.96 ± 0.35	2.68 ± 0.06	0.34

***A,B**. The tables show resting membrane potential, firing frequency (FF) and action potential properties in wild-type (wt) animals **(A)** or Panx1-ko mice **(B)** that were untreated, or received control (scrambled) peptide treatment*.

*AP, Action potential; AHP, afterhyperpolarization*.

### Aβ-Treatment Has Selective Effects on Intrinsic Excitability in Both Panx1-ko and Wild-Type Hippocampi

Having established that Panx1-ko mice exhibit higher intrinsic excitability compared to wild-type littermates, we now explored whether this property results in differing sensitivity to the effects of intracerebral oligomeric Aβ-treatment.

One week after Aβ-treatment, we detected no relative changes in resting membrane potential (Figure 2A) in wt (*N* = 6, *n* = 34) and Panx1-ko mice (*N* = 6, *n* = 34) compared to values detected in control peptide-treated animals. Input resistance increased in Aβ-treated wt compared to wt controls, but no change was evident in Aβ-treated wt compared to Panx1-ko controls (Figure 2B). No difference was detected in input resistance when Aβ-treated wt and Aβ-treated Panx1-ko were compared (Figure 2B). The excitatory threshold became elevated after Aβ-treatment in Panx1-ko mice compared to control ko (Figure 2C). No change in excitatory threshold was apparent in Aβ-treated wt, compared to wt controls (*p* = 0.2).

### Aβ-Treatment Accelerates Spike Frequency Adaptation and Reduces Action Potential Firing Frequency in Both Panx1-ko and Wild-Type Hippocampi

A comparison of spike frequency adaptation (SFA) in Aβ-treated vs. control-treated wt revealed significantly faster SFA in Aβ-treated wt hippocampi in the range of 300–1,000 ms (500 ms excepted, Figure 3A, Table 3). SFA in Aβ-treated Panx1-ko hippocampi was also significantly faster compared to control Panx1-ko hippocampi (Figure 3A, Table 3) in the range of 200 ms through 1,000 ms (300 ms excepted, Figure 3A, Table 3). SFA was significantly poorer in Aβ-treated wt mice compared to Aβ-treated Panx1-ko, but effects were limited to current injections at 400 and 600–800 pA (Figure 3A, Table 3). Taken together, these data suggest that absence of Panx1 accelerates SFA under control conditions. Furthermore, Panx1-ko limits the debilitating effects of Aβ-treatment on SFA, whereby Aβ-mediated enhancements in SFA correspond to the following hierarchy:

**Table 3:** Spike Frequency Adaptation (Hz) in wild-type and Panx1-ko hippocampi after treatment with oligomeric Aβ (1-42) or control peptide.

**Time during current injection**	**wt control**	**wt Aβ**	**Panx1-ko control**	**Panx1-ko Aβ**	**wt control vs. wt Aβ**	**Panx1-ko control vs. Panx1-ko Aβ**	**wt control vs. Panx1-ko control**	**wt Aβ vs. Panx1-ko Aβ**
100 ms	37.65 ± 2.24	41.18 ± 1.83	42.06 ± 2.79	38.53 ± 2.39	0.13	0.13	0.06	0.25
200 ms	24.71 ± 1.14	21.18 ± 1.73	28.24 ± 1.71	23.53 ± 1.68	0.13	**0.04**	0.12	0.30
300 ms	21.18 ± 0.92	15.88 ± 1.85	23.82 ± 1.52	20 ± 1.52	**0.02**	0.097	0.25	0.07
400 ms	18.53 ± 1.20	11.76 ± 1.61	22.94 ± 1.82	18.24 ± 1.66	**0.003**	**0.04**	0.06	**0.005**
500 ms	15.88 ± 1.20	12.06 ± 1.57	20.29 ± 1.49	15.59 ± 1.59	0.097	**0.04**	0.06	0.13
600 ms	15.29 ± 1.42	7.94 ± 1.45	19.41 ± 1.89	12.94 ± 1.49	**0.002**	**0.005**	0.07	**0.03**
700 ms	12.94 ± 1.30	7.35 ± 1.48	19.12 ± 1.66	12.35 ± 1.69	**0.02**	**0.003**	**0.008**	**0.03**
800 ms	11.47 ± 1.34	6.47 ± 1.46	16.47 ± 1.83	11.18 ± 1.78	**0.03**	**0.02**	**0.03**	**0.04**
900 ms	11.18 ± 1.25	5.29 ± 1.28	15.88 ± 1.47	8.82 ± 1.39	**0.01**	**0.002**	**0.04**	0.12
1,000 ms	10.88 ± 1.15	5 ± 1.28	14.41 ± 1.85	8.82 ± 1.68	**0.01**	**0.01**	0.12	0.097

*Spike frequency adaptation during current injections in the range of 100 through 1,000 ms are shown in the table. Effects are shown for wt mice that were treated with control (scrambled) peptide or oligomeric Aβ (leftmost columns) and for Panx1-ko mice that were treated with control peptide or oligomeric Aβ (3rd and 4th columns from the left). The four columns on the right show post-hoc Fisher's LSD p-values for comparisons of Panx1-ko, or wt animals that were treated with control peptide or Aβ, as a comparison of Panx1-ko and wt responses to control peptide, or to oligomeric Aβ. Mixed ANOVA for all values: F_(27, 1188)_ = 1.8940, p = 0.00389. Significant effects are highlighted in bold*.

Aβ-treated wt > Aβ-treated Panx1-ko = wt control > Panx1-ko control.

Firing frequency was significantly decreased in both Aβ-treated wt (Figures 3B,D) and Panx1-ko (Figures 3B,D). (ANOVA control wt vs. Aβ-treated wt: *F*_(3, 132)_ = 7.98, *p* = 0.00006, *post-hoc* LSD for 250 pA: *p* = 0.01, 300 pA: *p* = 0.003, 350 pA *p* = 0.0004, 400 pA *p* = 0.00005, ANOVA control Panx1-ko vs. Aβ-treated Panx1-ko: *F*_(3, 132)_ = 7.98, *p* = 0.00006, *post-hoc* LSD for 150 pA: *p* = 0.02, 200 pA: *p* = 0.004, 250 pA: *p* = 0.003, 300 pA: *p* = 0.001, 350 pA: *p* = 0.002, 400 pA: *p* = 0.004).

When all conditions were compared (Figure 3B), we found that control peptide-treated Panx1-ko showed the highest firing frequency, that was significantly different from firing frequency in control peptide-treated wt (*post-hoc* LSD for 100 pA: *p* = 0.009, 150 pA: *p* = 0.03, 200 pA: *p* = 0.02, 250 pA: *p* = 0.02, 300 pA: *p* = 0.008, 350 pA: *p* = 0.03). The decrease in firing frequency in Aβ-treated Panx1-ko hippocampi was indistinguishable from the firing frequency profile of control peptide-treated wt mice (Figure 3B).

### Action Potential Properties Are Unaltered in Wild-Type and Knockout Hippocampi 1 Week After Aβ-Treatment

One week after Aβ-treatment, further action potential properties were unaltered in both wt and Panx1-ko hippocampi (Figure 4). Thus, time-to-peak (Figure 4A), time from peak to the AHP (Figure 4B), the total spike time (Figure 4C), the ascending and descending slopes of the action potential (Figures 4D,E), the action potential half-width and 20%-width (Figures 4F,G) and the action potential peak (Figure 4H) remained unchanged in wt and Panx1-ko hippocampi compared to their respective controls. Some of the significant differences between these action potential peoperties in wt compared to Panx1-ko were sustained (time-to-peak (Figure 4A, *post-hoc p* = 0.01), total spike time (Figure 4C, *post-hoc p* = 0.04) and peak of AP (Figure 4H, *post-hoc p* = 0.02) after Aβ-treatment. For all other AP properties, there were no significant differences between wt and Panx1-ko after treatment with Aβ: time from peak to the AHP (*post-hoc p* = 0.08), the ascending (*post-hoc p* = 0.06) and descending (*post-hoc p* = 0.06) slopes of the action potential, the action potential half-width (*post-hoc p* = 0.06) and AP 20%-width (*post-hoc p* = 0.14) were all unchanged (Table 4).

**Table 4:** Action potential and afterhyperpolarization properties in wild-type and Panx1-ko hippocampal neurons after treatment with oligomeric Aβ(1-42) or control peptide.

	**wt control**	**wt Aβ**	**Panx1-ko control**	**Panx1-ko Aβ**	**ANOVA**
AP threshold (mV)	−38.26 ± 0.89	−38.75 ± 0.56	−37.01 ± 1.06	−36.79 ± 1.03	*p* = 0.36
Spike amplitude (mV)	96.52 ± 0.91	96.45 ± 0.90	97.00 ± 1.36	96.63 ± 1.32	*p* = 0.99
Time to peak (ms)	0.43 ± 0.01	0.43 ± 0.01	0.39 ± 0.01	0.39 ± 0.01	***p*** **=** **0.003**
Time peak to AHP (ms)	2.60 ± 0.06	2.57 ± 0.08	2.29 ± 0.06	2.41 ± 0.05	***p*** **=** **0.003**
Total spike time (ms)	3.03 ± 0.07	2.99 ± 0.08	2.68 ± 0.06	2.80 ± 0.05	***p*** **=** **0.001**
Ascending slope (mV/ms)	231.46 ± 7.44	230.83 ± 6.56	254.56 ± 8.20	251.49 ± 8.01	***p*** **=** **0.047**
Descending slope (mV/ms)	41.08 ± 1.18	41.93 ± 1.30	48.60 ± 1.37	45.40 ± 1.25	***p*** **=** **0.0002**
Half-width (ms)	0.97 ± 0.02	0.95 ± 0.02	0.86 ± 0.02	0.89 ± 0.02	***p*** **=** **0.0005**
20%-width (ms)	1.45 ± 0.03	1.40 ± 0.03	1.28 ± 0.03	1.34 ± 0.02	***p*** **=** **0.0004**
AP peak (mV)	58.26 ± 0.49	57.70 ± 0.75	59.99 ± 0.68	59.84 ± 0.67	***p*** **=** **0.035**
fAHP (mV)	−8.01 ± 0.72	−7.90 ± 0.40	−11.93 ± 0.90	−10.68 ± 0.73	***p*** **=** **0.0001**
mAHP (mV)	−4.86 ± 0.24	−5.58 ± 0.30	−4.74 ± 0.23	−5.42 ± 0.26	*p* = 0.062
sAHP (mV)	−1.43 ± 0.15	−2.05 ± 0.15	−1.40 ± 0.16	−1.90 ± 0.17	***p*** **=** **0.006**

*The table describes values obtained in assessing action potential (AP) and afterhyperpolarization properties of wt and Panx1-ko hippocampi that were treated with control (scrambled) peptide or Aβ*.

*AP, Action potential; AHP, afterhyperpolarization; fAHP, fast AHP; mAHP, medium AHP; sAHP, slow AHP*.

*Significant effects are highlighted in bold*.

### The Afterhyperpolarization Is Altered in Wild-Type and Knockout Hippocampi 1 Week After Aβ-Treatment

We observed that despite the fact that most passive membrane properties were unaltered in wt and Panx1-ko hippocampi 1 week after Aβ-treatment, firing frequency was significantly decreased in both animal cohorts (Table 5). We wondered if this could be explained by changes in the afterhyperpolarization elicited by Aβ-treatment. We observed a significant difference in the fast AHP in Aβ-treated wt and Panx1-ko hippocampi (Figure 5A), although effects with regard to the medium AHP were equivalent (Figure 5B). The slow AHP was more negative in both wt and Panx1-ko hippocampi after Aβ-treatment (Figure 5C), compared to their respective controls, but effects were not different between Aβ-treated wt and Panx1-ko hippocampi (Table 4). This suggests that intrinsic changes in the AP firing frequency may be mediated by Aβ-actions on the slow AHP, whereas differences between the firing frequency sensitivity of Aβ-treated wt and Panx1-ko hippocampi are mediated by Aβ-actions on the fast AHP (Table 4).

**Table 5:** Summary of effects of oligomeric Aβ (1-42) or control peptide-treatment on passive and active neuronal membrane properties of hippocampal pyramidal cells.

	**Control vs. Aβ**		**Control vs. control**	**Aβ vs Aβ**
	**wt × wt**	**ko × ko**	**wt × ko**	**wt × ko**
Resting potential	-	-	↓	-
Input resistance	↑	-	↑	-
Excitatory threshold	-	↑	↓	**-**
Firing frequency	↓	↓	↑	↑
Time to peak of AP	-	-	↓	↓
Peak to AHP	-	-	↓	**-**
Total spike time	-	-	↓	↓
Ascending slope	-	-	↑	**-**
Descending slope	-	-	↑	**-**
Half-width of AP	-	-	↓	**-**
20% width of AP	-	-	↓	**-**
AP peak	-	-	↑	↑

*The table summarises significant differences detected in Panx1-ko and wt mice after treated with oligomeric Aβ or control (scrambled) peptide. The outcome of statistical comparisons of control peptide vs. Aβ treatment of either Panx1-ko or wt mice (leftmost columns), control peptide effects in wt vs. Panx1-ko (middle column), and Aβ effects in wt vs. Panx1-ko (rightmost column) are summarised. In all cases, the upward-pointing arrows indicate an increase, and the downward-pointing arrows indicate a decrease in values in the control condition. A dash signifies no effect*.

*AHP, Afterhyperpolarization; AP, Action potential*.

### Panx1-ko Mice Are Resistant to Aβ-Mediated Deficits in Hippocampal Long-Term Potentiation

**Figure 6:**
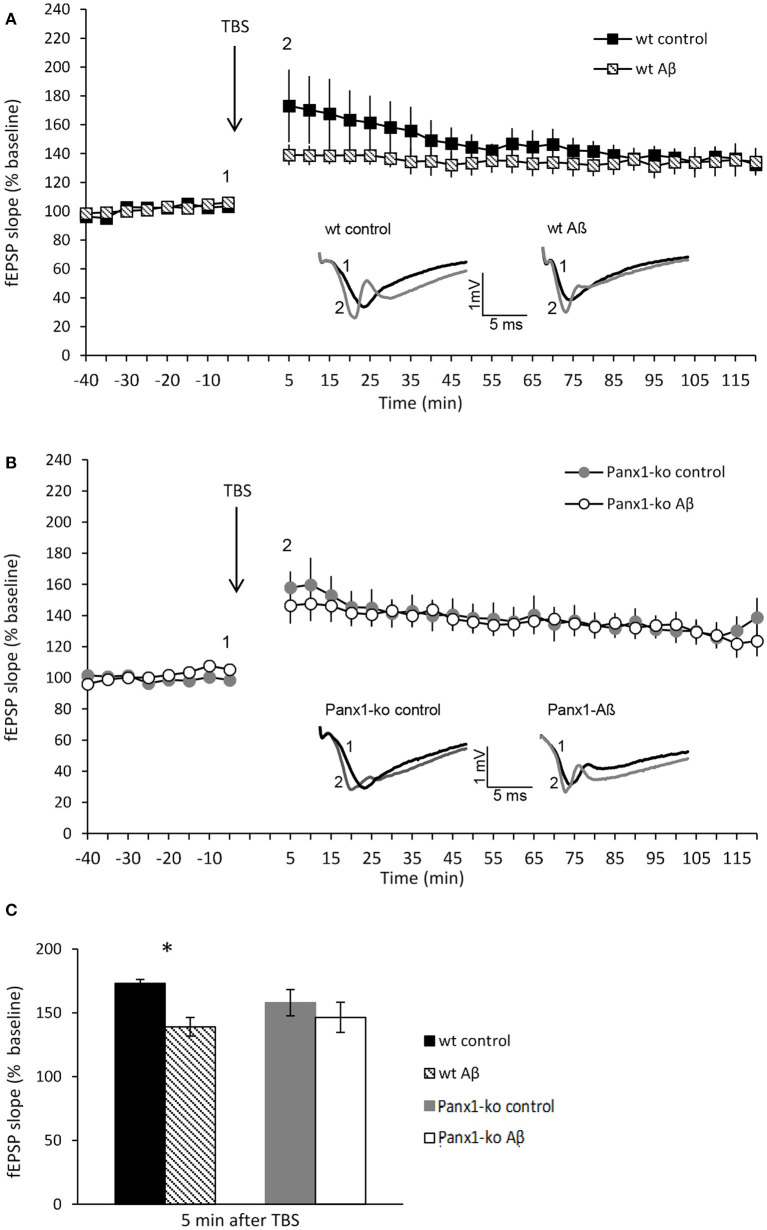
Panx1-ko mice are resistant to Aβ-mediated deficits in hippocampal long-term potentiation. **(A)** One week after Aβ-treatment, the early phase of hippocampal LTP was significantly impaired compared to control (scrambled) peptide-treated controls. **(B)** By contrast, LTP in Panx1-ko hippocampi was unaffected by Aβ-treatment compared to controls. **(C)** A comparison of fEPSP values evoked 5 min after TBS revealed a significant difference between control peptide-treated wt and Aβ-treated wt that was absent in equivalently treated Panx1-ko mice. Insets: analog examples of potentials evoked 5 min prior to theta burst stimulation (TBS) (1) and 5 min post-TBS (2), *p > 0.05.

Having detected specific changes in the action potential firing frequency and AHP following Aβ-treatment that were distinct in wt and Panx1-ko hippocampi, we wondered if these changes confer an advantage or a disadvantage in the enablement of hippocampal LTP. In wt mice we detected a significant impairment of the early phase of LTP [t-test revealed significance for the first 2 values (*p* = 0.002)] following Aβ-treatment (*N* = 6, *n* = 7) compared to control peptide treated wt (*N* = 6, *n* = 7) (Figure 6A). The later phases of LTP were unaffected by Aβ-treatment, however (Figure 6A).

By contrast, LTP was unaltered in Aβ-treated Panx1-ko hippocampi (*N* = 4, *n* = 6), compared to control peptide treated ko (*N* = 6, *n* = 9) (Figure 6B) [ANOVA 5 min-120 min post-TBS: *F*_(1, 11)_ = 0.19, *p* = 0.67]. A comparison of LTP evoked under control conditions in wt and Panx1-ko hippocampi revealed no significant differences (Figure 6C) [ANOVA 5 min-120 min post-TBS: *F*_(1, 14)_ = 0.62, *p* = 0.44].

This result suggests that the absence of Panx1 confers the hippocampus with a greater resistance to Aβ-mediated deficits in hippocampal long-term potentiation.

## Discussion

In this study, we observed that the hippocampi of Panx1-ko mice display higher neuronal excitability compared to their wildtype littermates, in line with previous findings (Prochnow et al., 2012). Firing frequency is particularly affected, with Panx1-ko hippocampi showing significantly higher responses both under control (scrambled) peptide and untreated conditions compared to wt-hippocampi. One week following intracerebral treatment with Aβ(1-42), both ko and wt hippocampi exhibited significant decreases in action potential firing frequency and increased negativity of the afterhyperpolarization. Strikingly, although LTP was impaired in Aβ-treated wt hippocampi, LTP was unaffected by Aβ-treatment in Panx1-ko, suggesting that the absence of Panx1 may be beneficial in the early stages of amyloidosis that accompany AD progression.

Our observation that multiple aspects of passive and active neuronal membrane properties were different in control peptide-treated Panx1-ko hippocampi, compared to control wt hippocampi, is consistent with reports by others that described increased neuronal excitability in Panx1-ko mice (Prochnow et al., 2012). In the present study, patch clamp recordings from CA1 pyramidal cells revealed a more positive resting membrane potential, a lower excitatory threshold, faster action potentials and a higher action potential firing frequency in control Panx1-ko hippocampi compared to control wt hippocampi. Furthermore, spike frequency adaptation was less effective in Panx1-ko hippocampi. Others have reported that the absence of Panx1 reduces seizure activity in an animal model of epilepsy (Santiago et al., 2011; Aquilino et al., 2020), indicating that Panx1 may be intrinsically involved in the maintenance of excitation in physiological ranges.

In animal models of AD that include aspects such as overexpression of amyloid precursor protein (APP), hyperexcitability has been reported from 3 to 4 months of age (Minkeviciene et al., 2009). Effects are mediated by inhibition of the GABA_A_ receptor (Orr et al., 2014). It has been proposed that the direct consequence of GABA_A_ receptor suppression will be increased extrasynaptic glutamate levels and an increase in the activation of GluN2B-containing NMDAR (Lei et al., 2016). In line with this, hippocampi of 3–4 week-old mice that were treated topically with oligomeric Aβ(1-40) demonstrated an increase in the number of population spikes triggered by a single stimulus, an increase in spontaneous EPSCs and a decrease in the number of IPSCs (Lei et al., 2016). The same authors found that pharmacological manipulations that normalized excitability, rescued Aβ(1-40)-mediated deficits in LTP. In our study, we did not detect changes in passive neuronal membrane properties consistent with changes in GABAergic or glutamatergic regulation in hippocampi from wt mice that had been treated 1 week previously with Aβ(1-42), although a significant increase in input resistance was evident in wt compared to control peptide-treated wt. These effects are consistent with a reduction in cell surface area and a reduced cation conductance of the cells (Rall, 1959; Spruston and Johnston, 1992). Action potential firing frequency was decreased and the slow afterhyperpolarization was more negative. The difference in our findings compared to the Lei et al., 2016 study may be related to differences in the mouse strain used, the age of the animals, the peptide used, or the treatment protocols. It may also be the case that an adaptation to the acute effects of Aβ-treatment occured in the week following inoculation, that was not possible in the short duration of the acute protocol followed by Lei et al. (2016).

In Aβ-treated Panx1-ko hippocampi, an increase in excitatory threshold occured compared to control peptide-treated ko mice. Firing frequency was decreased and spike frequency adaptation (SFA) was increased. The latter response profiles were similar to effects seen in control peptide–treated wt mice. Aβ-treated wt mice also exhibited a significant reduction of firing frequency compared to wt controls. The changes in firing frequency and SFA in both Aβ-treated Panx1-ko and wt mice were accompanied by changes in the afterhyperpolarization. In both cohorts we detected an increased negativity of the slow AHP, whereas in Panx1-ko mice we detected an additional increase in the negativity of the fast AHP. The fast AHP in both control and Aβ-treated Panx1-ko were significantly more negative than in control and Aβ-treated wt. These AHP changes may have formed the basis of the decreased firing frequency and alterations in spike frequency adaptation detected in both cohorts after Aβ-treatment. The findings in our wt animals are consistent with the observations of others with regard to changes in firing frequency and the AHP in the hippocampus after Aβ-treatment (Yun et al., 2006), or in transgenic mouse models of AD (Kaczorowski et al., 2011; Zhang et al., 2015). A low amplitude of the slow AHP lowers the threshold of LTP induction (Cohen et al., 1999; Kaczorowski and Disterhoft, 2009; Sehgal et al., 2013). Thus, the increased negativity of the slow AHP may have contributed to the significant impairment of LTP that we detected in Aβ-treated wt hippocampi. The question then arises as to why LTP was unaffected by Aβ-treatment in Panx1-ko hippocampi.

It has been reported by others that the blockade of Panx1-hemichannels causes a frequency-dependent shift in synaptic plasticity that favours LTP to the disadvantage of LTD and lowers the threshold for the induction of synaptic plasticity (Ardiles et al., 2014). Effects are associated with deficits in spatial reversal learning (Gajardo et al., 2018), a process that involves the updating of memory of spatial content that is tightly linked to hippocampal LTD (Kemp and Manahan-Vaughan, 2004, 2008; Goh and Manahan-Vaughan, 2013; Manahan-Vaughan, 2017). Furthermore, Panx1-ko mice also have deficits in object recognition memory (Prochnow et al., 2012), a phenomenon that is also tightly related to hippocampal LTD (Goh and Manahan-Vaughan, 2013). A shift from LTD to LTP in Panx1-ko mice may mean that the animals should show improvements in information acquisition that is associated with LTP, such as learning about global changes in space (Kemp and Manahan-Vaughan, 2007; Manahan-Vaughan, 2017). This may derive from changes in attention triggered by the absence of Panx1. In line with this, an increase in behavioral activity through the light-dark cycle has also been reported in Panx1-ko mice (Kovalzon et al., 2017). Increased wakefulness may have the downside of reduced sleep consolidation of acquired memories, however. Taken together, frequency-dependent shift in Panx1-ko mice towards LTP is a likely explanation for the resilience we observed in Aβ-treated Panx1-ko hippocampi against debilitations of LTP.

The shift in preference of LTP over LTD induction may be mediated by changes in the GluN2 subunit composition of NMDAR (Gajardo et al., 2018). Whereas GluN2B-containing NMDAR enable the induction of robust and lasting forms of LTP, GluN2A-containing NMDAR support the induction of weaker and more transient forms (Ballesteros et al., 2016). This would explain the failure of reversal learning in Panx1-ko mice: robust LTP is induced during cumulative spatial learning that becomes resistant to updating during reversal learning. Where does this possibility leave us in terms of our finding the Panx1-ko rescues Aβ-mediated deficits in LTP? Functionally, this should mean that spatial learning is reinforced, but it may also mean that flexible updating of acquired memories, or extinction learning is more difficult. Given that spatial memory loss is a distressing early symptom of AD, this might be a minor “trade-off” in a therapeutic approach that suppresses Panx1 function, such that LTP can be maintained at pre-symptomatic levels.

To what extent the restoration of LTP can be sustained is currently unclear. Mast cells are postulated to act as sensors to Aβ-accumulation and help propagate a neuroinflammatory response that attempts to protect against amyloidosis in AD (Harcha et al., 2015). Aβ triggers rapid mast cell degranulation thereby debilitating the mast cell response, but inhibition of Panx1 prevents this process (Harcha et al., 2015). Thus, inhibition of Panx1 might serve to maintain neuroprotective processes that in turn sustain hippocampal function at least in the early phases of AD. Consistent with this proposal, others have shown in a transgenic mouse model of AD, where APP and presenilin1 are overrexpressed, that blockade of Panx1 ameliorated synaptic defects in 6 months old APP/presenilin1 tg mice, and normalized LTP (Flores-Muñoz et al., 2020). Interestingly, this study also reported increased Panx1 expression in APP/presenilin1 tg mice suggesting that changes in Panx1 may contribute to the progress of AD.

## Conclusions

In this study we provide novel evidence that absence of Panx1 causes an increase in neuronal excitability and of action potential firing frequency, as well as a reduction of SFA in the CA1 region of the hippocampus compared to wt controls. We also provide evidence of early adaptation of the hippocampus to acute hyperexcitability effects of Aβ that have been reported by others (Lei et al., 2016): 1 week following intracerebral inoculation with Aβ(1-42) we detected no generalized changes in neuronal excitability in ko and wt compared to their respective controls. However, afterhyperpolarization responses became more negative, firing frequency was reduced and SFA enhanced in both Aβ-treated Panx1-ko and wt hippocampi. In addition the excitatory threshold was lower in Panx1-ko, suggesting that early adaptation to Aβ(1-42) involves a *reduction* of neuronal excitability. Strikingly early LTP was significantly impaired in Aβ-treated wt, but was unaffected in Panx1-ko. These data suggest that the absence of Panx1 provides the hippocampus with increased resilience to the debilitating effects of oligomeric Aβ(1-42).

## Data Availability Statement

The raw data supporting the conclusions of this article will be made available by the authors, upon reasonable request.

## Ethics Statement

The animal study was reviewed and approved by Landesamt für Arbeitsschutz, Naturschutz, Umweltschutz und Verbraucherschutz, Nordrhein Westfalen, Germany.

## Author Contributions

The study was designed by DM-V. NS, and OS and conducted experiments, analysis, and paper writing. All authors contributed to the article and approved the submitted version.

## Conflict of Interest

The authors declare that the research was conducted in the absence of any commercial or financial relationships that could be construed as a potential conflict of interest.

## Supplementary Material

Supplementary Figure 1 Untreated Panx1-knockout mice exhibit higher action potential firing frequency compared to wild type mice. Action potential firing frequency was significantly faster in untreated Panx1-ko mice compared to untreated wild-type (wt) animals [ANOVA: *F*_(1, 40)_ = 4.9812, *p* = 0.03129].
