# Supplementary figures and images for: Absence of Pannexin 1 Stabilizes Hippocampal Excitability After Intracerebral Treatment With Aβ (1-42) and Prevents LTP Deficits in Middle-Aged Mice
Source: Front Aging Neurosci. 2021 Mar 16;13:591735. doi: 10.3389/fnagi.2021.591735 (PMC8007872; doi:10.3389/fnagi.2021.591735)

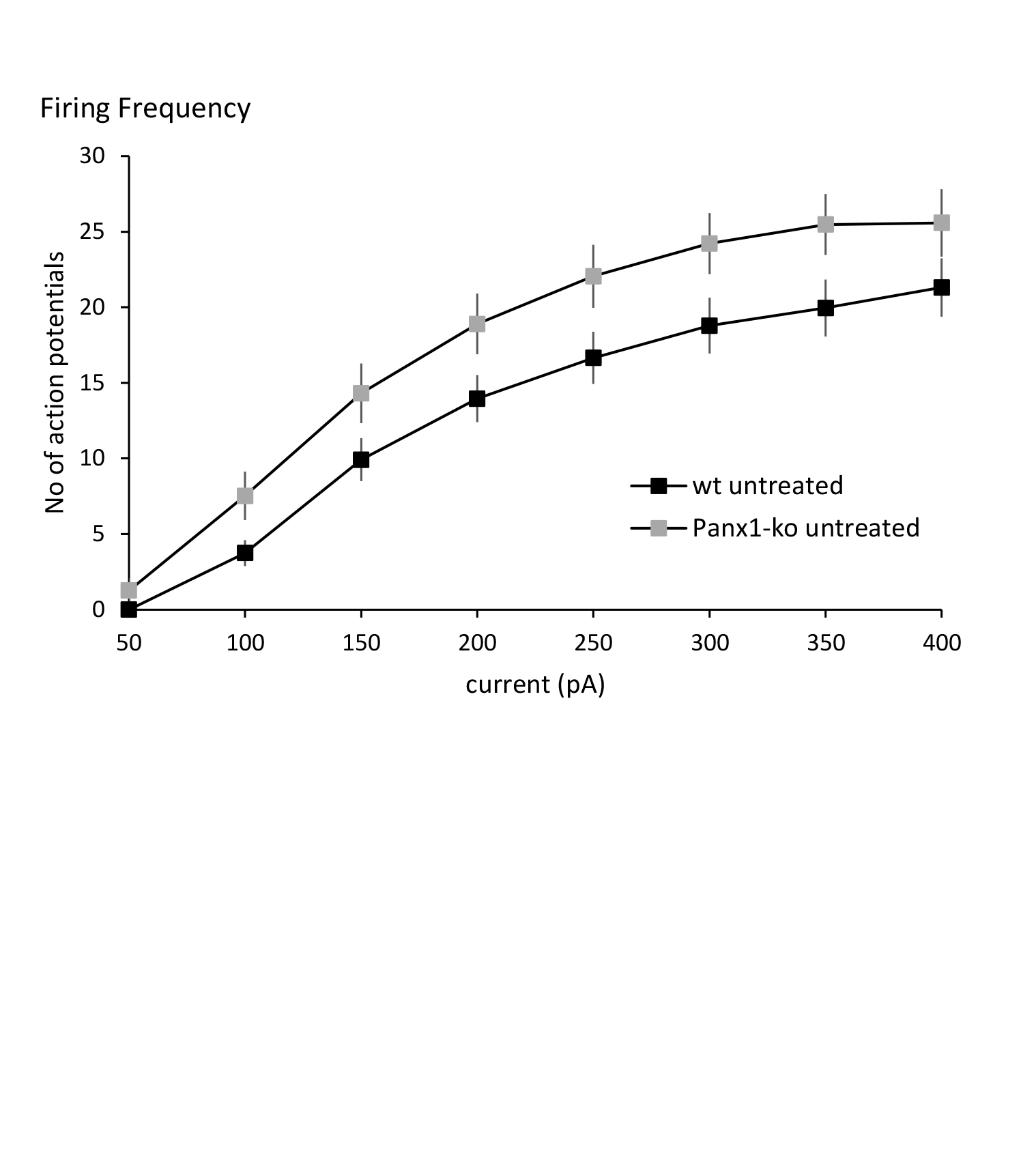

Supplement: Supplementary Figure 1 — Untreated Panx1-knockout mice exhibit higher action potential firing frequency compared to wild type mice. Action potential firing frequency was significantly faster in untreated Panx1-ko mice compared to untreated wild-type (wt) animals [ANOVA: F(1, 40) = 4.9812, p = 0.03129]. [file Image_1.TIF]
